# Bevacizumab combined with chemotherapy for ovarian cancer: A protocol for systematic review and meta-analysis

**DOI:** 10.1097/MD.0000000000028376

**Published:** 2021-12-23

**Authors:** Ting Liao, Li Li, Liya Wang

**Affiliations:** a Department of Obstetrics and Gynecology, Chongqing General Hospital, Chongqing, China; b Department of Gynecology, Zhongxian People's Hospital of Chongqing, Chongqing, China; c Department of Obstetrics and Gynecology, the Sixth People's Hospital of Chongqing, Chongqing, China.

**Keywords:** chemotherapy, meta-analysis, ovarian cancer, survival

## Abstract

**Background:**

The impact of bevacizumab (an anti-vascular endothelial growth factor therapy) remains uncertain, which has been the focus of studies on the management of ovarian cancer (OC). We performed a protocol for systematic review and meta-analysis to assess the efficacy and safety of bevacizumab combined with chemotherapy in OC.

**Methods:**

The presentation of methods and results in this systematic review was performed according to the evaluation guidelines for health care interventions provided in the Preferred Reporting Items for Systematic Reviews and Meta-Analyses Protocol. This study will use the Cochrane Library, Web of Science, PubMed, Embase, Allied and Complementary Medicine Database, China Biomedical Literature Database, China National Knowledge Infrastructure, China Science and Technology Journal Database, Wanfang Database, and Ongoing Clinical Trials Database. The risk of bias of included studies is estimated by taking into consideration the characteristics including random sequence generation, allocation concealment, blinding of patients, blinding of outcome assessment, completeness of outcome data, selective reporting, and other bias by Cochrane Collaboration's tool. All analyses were performed with Review Manager (RevMan) software, version 5.3.

**Results:**

The results of this systematic review and meta-analysis will be published in a peer-reviewed journal.

**Conclusion:**

Bevacizumab combined with chemotherapy may improve progression-free survival and overall survival in patients with OC.

## 1 Introduction

Ovarian cancer (OC) is one of the most debated oncologic disease in western and industrialized countries for different reasons.^[1,2]^ It is the second most common malignant gynecological disease, constantly showing an increase of incidence and prevalence; moreover among all the gynecological tumors it shows the main highest of mortality^[3]^; despite recent important advances in early diagnosis it is usually detected at a very advanced stage; finally its prognosis remains unsatisfactory even today, with poor survival rates, despite the current availability of many active and emerging drugs.

There is also great debate and confusion about who is the specialist appointed to manage the patient with OC; often this uncertainty implies that OC is frequently treated in centers not sufficiently skilled by a multidisciplinary point of view. The recognized standard treatment for OC is aggressive cytoreductive surgery (when it is possible), followed by a chemotherapeutic association of platinum and taxanes, according to different schedules.^[4]^ Even if this first line chemotherapy is highly active, with objective response rate >75%, a large number of patients recur or experience cancer progression, finally dying for this disease.

The formation of new blood vessels, known as angiogenesis, is essential for solid tumor growth and metastasis.^[5,6]^ Vascular endothelial growth factor (VEGF) is a protein-signaling molecule that acts on endothelial cells to promote angiogenesis.^[7,8]^ VEGF is overexpressed in epithelial OC making targeting of angiogenesis through the VEGF-signaling pathway an attractive therapeutic option.^[9,10]^ Bevacizumab is an anti-VEGF antibody that has demonstrated activity in OC. Several randomized controlled trials (RCTs) have been launched to evaluate the efficacy and safety of bevacizumab combined with chemotherapy in OC. However, the survival benefit of bevacizumab was different in these trials.^[11,12]^ Thus, we performed a protocol for systematic review and meta-analysis to assess the efficacy and safety of bevacizumab combined with chemotherapy in OC.

## 2 Method

### 2.1 Study registration

The protocol of this review was registered in OSF (OSF registration number: 10.17605/OSF.IO/MNZQJ). It is reported to follow the statement guidelines of preferred reporting items for systematic reviews and meta-analyses protocol.^[13]^ Since this study is on the basis of published studies, ethical approval is not required.

### 2.2 Searching strategy

This study will use the Cochrane Library, Web of Science, PubMed, Embase, Allied and Complementary Medicine Database, China Biomedical Literature Database, China National Knowledge Infrastructure, China Science and Technology Journal Database, Wanfang Database, and Ongoing Clinical Trials Database. There is no definite time limit for the retrieval literature, and the languages are limited to Chinese and English. We will consider articles published between database initiation and December 2021. The following Medical Subject Headings (MeSH) terms and free text were used: “ovarian cancer,” “Bevacizumab,” and “randomized controlled trial.”

### 2.3 Inclusion and exclusion criteria

The criteria for inclusion were as follows: prospective RCTs studying bevacizumab in patients with OC, bevacizumab added as maintenance therapy after chemotherapy, or concurrently with chemotherapy followed by a maintenance period, RCTs reporting the numbers of patients with hazard ratios (HRs) and 95% confidence intervals (CIs) of progression-free survival and overall survival (OS) or presenting sufficient data for calculating HRs with 95% CIs, RCTs reporting the numbers of patients with toxicity or adverse events, and language limited to English or Chinese.

The exclusion criteria for this study were as follows: conference abstracts, case reports, comments, editorials, etc; for multiple publications that have been determined to be reported in the same clinical study, the publication with the most complete publication data is qualified; and the literature information was insufficient to extract the required useful data.

### 2.4 Study selection

Two authors independently reviewed all titles and abstracts of studies identified by the above searches. Full texts of any potentially useful studies were reviewed, and disagreements were resolved by discussion.

### 2.5 Data extraction

The following data were extracted for each article: bibliographical data, including authors and year of publication; clinical trial features such as sample size, study flow, recruitment method, criteria for inclusion and exclusion, primary measures, time and point of assessments, and duration of the intervention; participant characteristics such as age, sex, and so on; patient background, including country and race; and study drop-out rate and handling of missing data.

### 2.6 Risk of bias assessment

Two investigators will separately assess the risk of bias of the included studies using the Cochrane risk of bias assessment tool. The evaluation of each study mainly included the following 7 aspects: random sequence generation, allocation hiding, blinding of participants and personnel, blinding of outcome assessment, incomplete outcome data, selective outcome reporting, and other biases. Finally, the bias of the study will be rated on 3 levels: “low,” “high,” and “ambiguous.” These even domains will be separately appraised by 2 reviews, and discrepancies will be addressed by consulting a third reviewer.

### 2.7 Statistical analysis

Statistical heterogeneity was explored using a chi-square test and expressed as an *I*^2^ index (a significance level of *P* < .10). *I*^2^ values over 50% represented substantial heterogeneity. If there was no heterogeneity, a fixed-effects model was used for meta-analysis; otherwise, a random-effects model based on the DerSimonian and Laird estimator was used.^[14]^ HRs and their 95% CIs of all the included trials for time-to-event data for generic inverse variance data were calculated by determining the weighted average of individual study results. A 2-sided *P* < .05 was considered statistically significant. Potential publication bias was tested with a funnel plot. To evaluate publication bias, we will construct a funnel plot if the number of included studies is sufficient. All analyses were performed with Review Manager (RevMan) software, version 5.3 (Update Software Ltd, Oxford, Oxon, UK).

## 3 Discussion

The standard treatment of OC since the 1990s has been a combination of platinum and taxane. Since then, only marginal improvements have been made, in variations to chemotherapy dosing, scheduling, and the route of administration, in either a frontline or a recurrent setting.^[15]^ Angiogenesis plays a critical role in normal ovarian physiology, as well as in the pathogenesis of OC. VEGF is the best-characterized angiogenic factor and is recognized as a major element in regulating angiogenesis.^[16]^ Increased VEGF expression has been associated with the development of tumor progression and poor overall survival.^[17]^ As the first approved anti-VEGF drug for treatment of OC by the US Food and Drug Association, bevacizumab is the most widely studied. This meta-analysis was to assess the efficacy and safety of bevacizumab combined with chemotherapy in OC. Heterogeneity is inevitable due to different patient populations, lines of treatment, doses of bevacizumab, concurrent chemotherapies, follow-up durations, and lengths of bevacizumab maintenance. More high quality RCTs were still required.

## Author contributions

**Conceptualization:** Li Li.

**Data curation:** Li Li.

**Funding acquisition:** Liya Wang.

**Investigation:** Li Li.

**Methodology:** Ting Liao.

**Writing – original draft:** Ting Liao.

**Writing – review & editing:** Liya Wang.
